# Changes of peripheral TGF-β1 depend on monocytes-derived macrophages in Huntington disease

**DOI:** 10.1186/1756-6606-6-55

**Published:** 2013-12-13

**Authors:** Alba Di Pardo, Silvia Alberti, Vittorio Maglione, Enrico Amico, Etty P Cortes, Francesca Elifani, Giuseppe Battaglia, Carla L Busceti, Ferdinando Nicoletti, Jean Paul G Vonsattel, Ferdinando Squitieri

**Affiliations:** 1 IRCCS Neuromed, 86077 Pozzilli, Italy; 2 Department of Pathology, College of Physicians and Surgeons, Columbia University, 10032 New York, USA; 3 Department of Human Physiology and Pharmacology, University “Sapienza”, 00185 Rome, Italy

**Keywords:** Cytokines in Huntington disease, TGF-β1, Monocytes-derived macrophages, Macrophages polarization

## Abstract

**Background:**

Huntington Disease (HD) is a neurodegenerative disorder resulting from the expansion of polyglutamine stretch in the huntingtin protein (Htt). Mutant HTT (mHtt) leads to progressive impairment of several molecular pathways that have been linked to disease pathogenesis. Defects in the production of a number of neurotrophic factors have been described as important determinants contributing to the development of HD. We have previously demonstrated that production of transforming growth factor-β1 (TGF-β1) is also deregulated in HD. Peripheral levels of TGF-β1 were markedly reduced early in the disease and returned to normal levels with disease severity. However, the cause and the biochemical origin of such abnormalities are still unclear.

**Results:**

We report here that the abnormal production of peripheral TGF-β1 depends on the changes in the percentage of TGF-β1-producing macrophages along disease course. Variation in the number of TGF-β1-producing macrophages resulted from differential activation state of the same cells, which displayed phenotypic and functional heterogeneity throughout the clinical course of HD. We further demonstrated that, similar to the periphery, the number of TGF-β1-immunoreactive cells in human post-mortem brain with HD, varied with neuropathological changes.

**Conclusions:**

Our data indicate that reduced bioavailability of TGF-β1 in the serum of HD subjects is attributable to the variation of the number of TGF-β1-producing macrophages. Macrophages display a differential ability to produce TGF-β1, which reflects diversity in cells polarization throughout the disease course. Besides elucidating the biochemical origin of TGF-β1 fluctuations in HD, our study highlights an interesting parallelism between periphery and central compartment and underlines the potential of TGF-β1 as a possible indicator suitable for prediction of disease onset in HD.

## Background

Huntington disease (HD) is a progressive neurodegenerative disorder, caused by an expanded CAG repeat within *HTT* gene encoding an abnormal long polyglutamine (polyQ) stretch in the huntingtin protein (Htt). Elongated polyQ tract contributes to either gain-of-toxic function of Htt or loss-of-function of many other proteins, resulting in a broad array of cell dysfunctions within and out the nervous system [1]. In the brain, progressive striatal atrophy, degeneration of cortico-striatal fibers and glial activation are characteristic features of HD and represent early events in the disease course. Although the disease has traditionally been described as a disorder purely of the brain, abnormalities outside the central nervous system (CNS) are commonly found in HD [2]. Mutant huntingtin (mHtt) has been widely described to be highly expressed in immune cells which are becoming increasingly interesting in the study of neurodegenerative disorders as well as in the pathogenesis of the disease [3,4]. Defective regulation of growth factors, including brain-derived neurotrophic factor (BDNF) [5] and glial-derived neurotrophic factor (GDNF) [6] has been reported to affect CNS function [7] and to contribute to the pathogenesis of the disease [5,8]. Production of transforming growth factor-β1 (TGF-β1), a growth factor with established neuroprotective function and powerful anti-inflammatory properties [9] is also reported altered in HD [10]. Levels of TGF-β1 dynamically vary with HD development in both central and peripheral districts [10]. TGF-β1 plays a critical role in the regulation of several physiological processes including cell cycle control, cell differentiation and immune functions [11]. In addition to that, TGF-β1 contributes to maintain neuronal survival and integrity of CNS and regulates microglia activation [12]. Perturbations of the TGF-β1 signaling are involved in many neurodegenerative disorders [13]. An aberrant expression of TGF-β1 receptor II (TGFRII) has been reported in the brain of Alzheimer’s disease (AD) patients [14-17]. Reduced TGF-β1 signaling increases amyloid deposition and neurodegeneration in transgenic AD mice [13]. The role of TGF-β1 has been also investigated in several other neurodegenerative diseases such as Amyotrophic Lateral Sclerosis (ALS) [18], Parkinson disease (PD) and Prion diseases [9]. Reduced levels of TGF-β1 in the brain increase susceptibility to excitotoxic injury and neurodegeneration in heterozygous TGF-β1 knockout mice [12].

Under normal conditions, the expression of TGF-β1 is minimal and drastically up-regulates under pathologic circumstance, during which it plays a key role in the coordination of inflammatory responses and tissues recovery [19-21]. TGF-β1 is predominantly synthesized by neurons and glial cells, within the CNS, and by platelets and monocytes/macrophages in the peripheral tissues [22,23].

Macrophages display remarkable plasticity that enables them to perform distinct and even opposing function, such as release of either inflammatory or anti-inflammatory cytokines and growth factors, in response to different environmental cues [24]. Depending on the activation state, macrophages can be designed as either classical activated (M1), with pro-inflammatory properties, or alternatively activated (M2) cells, which mediate anti-inflammatory response [25]. Under physiological condition, macrophages, like glia, interact with their surroundings and provide protective cytokines and neurotrophins. Upon insult, both cell populations can become pathologically activated leading to neuro-inflammation, and/or neurodegeneration by altering expression of many neurotrophic factors [26].

In this study, we demonstrated that changes of peripheral TGF-β1 levels in HD depend on the variation in the percentage of TGF-β1-producing monocytes-derived macrophages along disease course. The differential capacity of macrophages to produce TGF-β1 reflects different cell phenotypes during the disease. After an early pro-inflammatory phenotype, macrophages switched towards an anti-inflammatory profile with disease progression. Although not completely elucidated, changes of nuclear factor-κB (NF-κB)-p65 expression/regulation may likely represent one of the molecular mechanisms governing macrophages heterogeneity in HD.

## Results

### Abnormal levels of peripheral TGF-β1 in HD depend on monocytic/macrophagic cell subset

**Figure 1:**
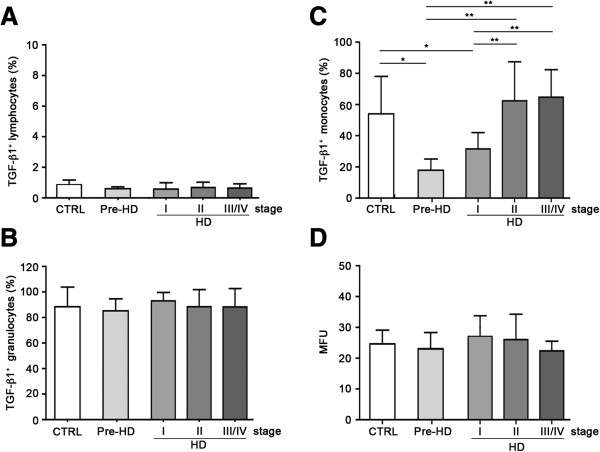
**pre-HD subjects and stage I HD patients showed lower percentage of TGF-β1-producing (TGF-β1**^**+**^**) monocytes compared to healthy control subjects and late stage HD patients. A** and **B**, Bar histograms showing the percentage of TGF-β1-producing lymphocytes and granulocytes in HD individuals (grey bars) and healthy control subjects (white bar). **C**, Bar histograms showing reduced percentage of TGF-β1^+^ monocytes in pre-manifested (pre-HD) subjects and stage I HD patients with respect to healthy controls (CTRL) and late stage HD patients (HD). **D**, Bar histograms showing similar intracellular TGF-β1 content (MFU) in monocytes from HD individuals (grey bars) and healthy control subjects (white bar). Data are shown as mean ± s.d. * *p* < 0.05 ** *p* < 0.001 (ANOVA, Tukey post-hoc test).

In order to identify what peripheral cell population primarily determined fluctuation of TGF-β1 levels in the serum of HD patients we examined the contribution of each whole blood cell subset at producing the cytokine by flow cytometry analysis (FACs). We first demonstrated similar absolute counts (n° cells ×10^3^/ml) of whole blood cell subsets (lymphocytes, monocytes and granulocytes) in HD individuals (Table 1) and controls (Additional file 1). Each cell subset was then examined to determine the relative contribution to TGF-β1 production. The percentage of lymphocytes and granulocytes producing TGF-β1 was similar in HD individuals and healthy controls (Figure 1A, B). Conversely, the percentage of TGF-β1-producing (TGF-β1^+^) monocytes was markedly low in pre-manifested (pre-HD) and clinical stage I subjects and gradually increased in advanced-stage HD (HD) patients up to control values (Figure 1C, Additional file 2). Analysis of TGF-β1 intracellular content, reported as mean fluorescence unit (MFU), in TGF-β1^+^ cells, did not differ significantly among all the groups (Figure 1D), suggesting no perturbation in the synthesis of the neurotrophin. On the other hand, immunoblotting analysis on total monocyte population showed that TGF-β1 protein expression varied coherently with the changes of the percentage of TGF-β1-producing monocytes along disease stages (Additional file 3).

**Table 1:** Demographic and clinical data of HD patients

	**Number (M,F)**	**Age (24-77 yrs)**	**TFC (13–0)**	**CAG repeats**[27-43]	**AO** [16-69]	**HD DI (yrs)**[40,41]	**DB (108–759)**	**DS (100–0)**	**UHDRS1 (1–100)**	**UHDRS2 (248–7)**	**UHDRS3 (0–88)**	**UHDRS4 (0–25)**	**MMSE (0–30)**
Pre-HD	14	35,4 ± 4,4	13	44,0 ± 2,4	-	-13,8 ± 7,9	296,9 ± 70,8	99,7 ± 0,9	5,8 ± 4,3	278,3 ± 33,1	6,2 ± 6,9	25,0 ± 0,0	27,8 ± 1,3
	(M = 8, F = 6)												
I HD	15 (M = 9, F = 6)	46,9 ± 8,4	11,8 ± 1,1	44,1 ± 2,2	44,2 ± 8,1	-1,6 ± 6,2	389,5 ± 70,2	92,1 ± 4,1	24,1 ± 7,9	186,6 ± 54,1	16,5 ± 7,5	23,7 ± 1,7	26,9 ± 1,7
II HD	36 (M = 17, F = 19)	55,4 ± 10,9	7,7 ± 0,8	43,2 ± 3,0	47,1 ± 10,3	3,9 ± 10,3	403,8 ± 124,1	77,2 ± 6,3	39,3 ± 12,2	132,2 ± 52	19,7 ± 7,9	17,6 ± 4,2	23,3 ± 4,5
III HD	31 (M = 18, F = 13)	53,6 ± 11,7	4,6 ± 1,3	44,5 ± 3,5	44,1 ± 10,1	6,7 ± 6,7	448,8 ± 110,1	61,3 ± 11,6	55,4 ± 12,8	89,1 ± 48,8	23,4 ± 10,0	9,8 ± 5,1	20,2 ± 5,0
IV HD	10 (M = 4, F = 6)	59,2 ± 10,5	1,6 ± 0,5	44,0 ± 3,6	46,4 ± 10,2	11,7 ± 6,1	470,7 ± 128,0	39,0 ± 8,4	75,8 ± 21,4	71,2 ± 44,9	23,3 ± 14,2	1,3 ± 0,8	21,0 ± 5,1
V HD	6 (M = 2, F = 4)	58,3 ± 13,4	0	45,0 ± 5,3	41,0 ± 15,6	10,3 ± 5,9	499,5 ± 127,0	22,9 ± 6,4	90,2 ± 13,4	-	15,0 ± 6,0	0,2 ± 0,4	11,4 ± 3,0

Control subjects are 46 (M = 19, F = 27; mean age: 49,3 ±10,7). Values are given as mean ± s.d.

yrs: years; TFC: total functional capacity; AO: age at onset; HD DI: Huntington Disease Development Index; DB: Disease Burden; DS: Diasability.

Score; UHDRS: Unified Huntington Disease Rating Scale; MMSE: Mini Mental State Examination.

**Figure 2:**
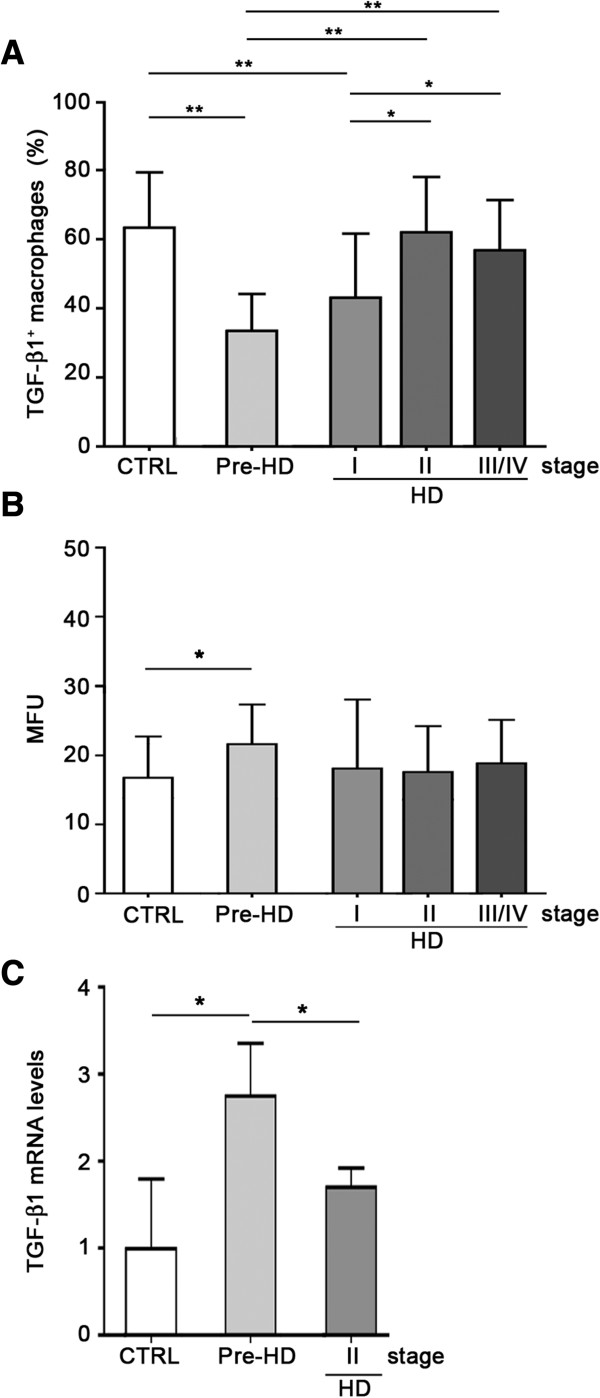
**Percentage of TGF- β1**^**+ **^**macrophages was reduced in pre-manifested subjects (pre-HD) and stage I HD patients. A**, Bar histograms showing reduced percentage of TGF-β1^+^ macrophages in pre-symptomatic (pre-HD) and stage I HD individuals compared to healthy controls (CTRL) and late stage HD patients (HD). **B**, Bar histograms showing increased intracellular TGF-β1 content, MFU, in macrophages from HD individuals (grey bars) and healthy control subjects (white bar). **C**, Bar histograms showing increased mRNA levels of TGF-β1 in pre-HD subjects compared to healthy controls (CTRL) and HD patients. Data are shown as mean ± s.d. * *p* < 0.05 ** *p* < 0.001 (ANOVA, Tukey post-hoc test).

Similar to monocytes, monocytes-derived macrophages from pre-HD subjects and clinical stage I patients showed only a small portion of TGF-β1^+^ cells when compared to cells derived from advanced HD stage patients and controls (Figure 2A, Additional file 4). No difference in the number of TGF-β1^+^ cells was detected between severe symptomatic patients and normal control subjects (Figure 2A), or between controls themselves (Additional file 5). MFU values relating to macrophages were significantly higher in pre-HD subjects as compared to controls (Figure 2B), indicative of an increased expression of intracellular TGF-β1 per cell at the early stage of the disease. Consistently, analysis of gene expression showed a robust increase of TGF-β1 mRNA levels in pre-HD compared either with control subjects or more advanced HD patients (Figure 2C). No difference in both MFU and TGF-β1 gene expression was detected between later stage HD patients and controls (Figure 2B,C).

### Macrophages display different degrees of polarization throughout HD course

**Figure 3:**
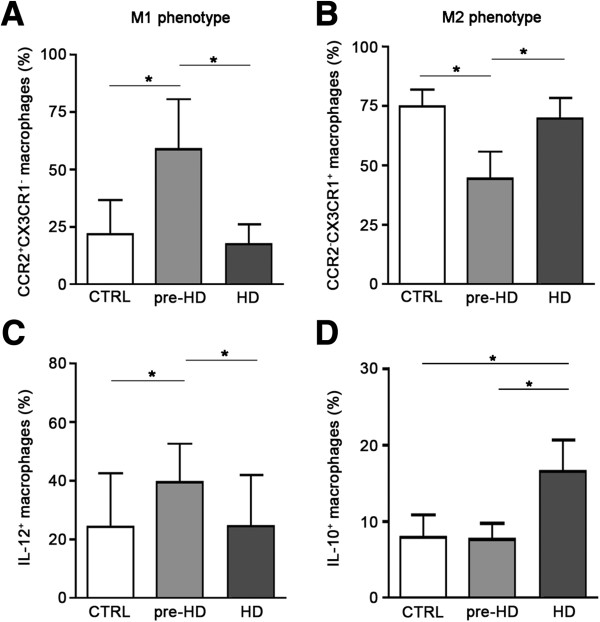
**Macrophages displayed different degrees of polarization during HD course. A** and **B**, Bar histograms showing variable percentage of CCR2^+^CX3CR1^-^ (M1) and CCR2^-^CX3CR1^+^ (M2) macrophages in pre-manifested subjects (pre-HD), severe HD patients (HD) and healthy controls (CTRL). **C** and **D**, Bar histograms showing changes in the percentage of IL-12 and IL-10 positive macrophages in pre-manifested subjects (pre-HD), severe HD patients (HD) and healthy control subjects (CTRL). **C**, IL-12-producing macrophages were increased in preHD subjects coherently with M1 phenotype. **D**, IL-10-producing macrophages were increased in HD patients, consistently with changes of M2 phenotype. Data are shown as mean ± s.d * *p* < 0.05 ** *p* < 0.001 (ANOVA, Tukey post-hoc test).

In an attempt to investigate whether dynamic change of TGF-β1-production during HD course was due to a phenotypic heterogeneity of macrophagic cells, we explored cell surface markers associated with either M1 or M2 phenotypes in monocyte-derived macrophages from HD individuals and healthy controls. Pre-HD subjects and clinical I stage HD patients showed a preferential pro-inflammatory M1 phenotype, - high percentage of CCR2^+^CX3CR1^-^ cells- and low percentage of CCR2^-^CX3CR1^+^ cells (Figure 3A). Macrophages from HD patients in the late stage of the disease, displayed changes in the expression of surface markers in favour of anti-inflammatory M2 phenotype - high frequency of CCR2^-^CX3CR1^+^ cells – and low percentage of CCR2^+^CX3CR1^-^ cells (Figure 3B). Expression of M1 and M2 surface markers was further confirmed by RT-PCR (data not shown).

Macrophages phenotype can be identified based also on the production of specific cytokines [27]. Consistent with phenotypic heterogeneity of macrophages in HD, we found that the percentage of pro-inflammatory IL-12- producing (IL-12^+^) cells (M1) was significantly increased early in the disease and returned to control values in the late stages HD patients (Figure 3C). Reduction in the frequency of IL-12^+^ cells in symptomatic HD patients, was associated with a concomitant increase in the percentage of anti-inflammatory IL-10-producing (IL-10^+^) macrophages (M2 phenotype) (Figure 3D). Despite such phenotypic diversity, however, the intracellular content of both IL-12 and IL-10 did not differ significantly among all the groups (data not shown).

### NF-κB pathway contributes to macrophages heterogeneity in HD

**Figure 4:**
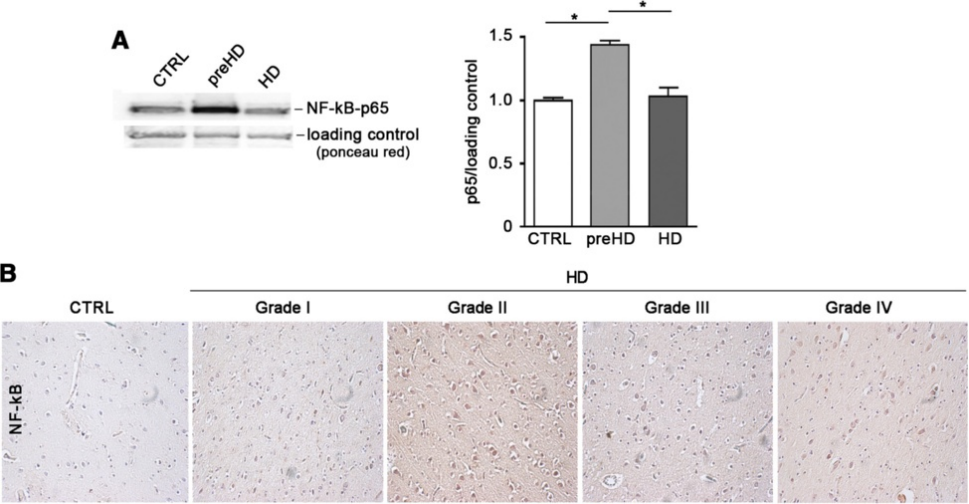
**NF-κB -p65 protein expression is increased in macrophages from preHD subjects. A**, Representative immunoblot (left) and densitometric analysis (right) of NF-κB -p65 expression in healthy controls (CTRL, n = 5), pre-manifested subjects (pre-HD, n = 5) and severe HD patients (HD, n = 5). Bars represent the mean values ± s.d *p < 0.05 (ANOVA, Tukey post-hoc test). **B**, Representative microphotographs showing NF-κB -p65 immunoreactivity on formalin-fixed and paraffin-embedded post-mortem striatal tissues from control subjects (CTRL) and HD patients at different neuropathological grades (Grade I, II, III, IV).

In order to clarify the possible molecular mechanism underlying the differential pattern of macrophages activation along disease course, we investigated the potential involvement of NF-κB in the promotion of distinct macrophage phenotypes. Analysis of protein expression indicated that monocytes-derived macrophages from pre-HD subjects displayed higher levels of NF-κB-p65 compared to symptomatic HD patients (Figure 4A). No differences were observed between healthy controls and symptomatic HD patients (Figure 4A). Interestingly, immunohistochemical staining for NF-κB-p65 in different graded postmortem brain tissues showed NF-κB-p65 expression changing profile similar to that observed in periphery (Figure 4B).

### **TGF-**β**1 levels in human HD post-mortem striatum change with disease-stages**

**Figure 5:**
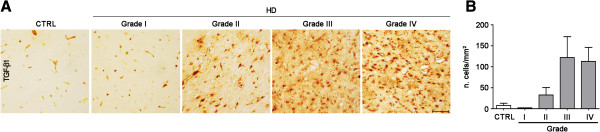
**Number of TGF-β1-producing cells in post-mortem brain tissues increased with pathological grade in HD patients. A**, Representative microphotograph showing TGF-β1 immunoreactivity on formalin-fixed and paraffin-embedded post-mortem striatal tissues from control subjects (CTRL) and HD patients at different neuropathological grades (Grade I, II, III, IV). **B**, Bar graph showing semi-quantitative analysis of TGF-β1 immunoreactive cells in HD brains. Data are presented as mean values ± s.d.

Immunohistochemical analysis in post-mortem human brain striatum, obtained from HD subjects and healthy controls, showed variation of the number of TGF-β1 immunoreactive cells during disease progression (Figure 5, Table 2) with a changing profile similar to that observed in the periphery. TGF-β1 immunoreactivity was first detected in pathological grade II HD brain tissues and gradually increased with disease severity reaching a peak in grade III-IV HD brains (Figure 5A, B).

**Table 2:** Pathological and clinical data of three control sublects and ten HD patients analysed for TGF-β1 expression in the striatum post-mortem sample

**Patients**		**Age**	**Gender**	**Vonsattel et al’s grade**
		**(years)**		
				[67]
1	Healthy control	74	M	CTRL
2	Healthy control	67	M	CTRL
3	Healthy control	79	F	CTRL
4	Symptomatic	69	M	I
5	Symptomatic	50	M	II
6	Symptomatic	58	M	II
7	Symptomatic	69	M	II
8	Symptomatic	53	M	III
9	Symptomatic	56	M	III
10	Symptomatic	53	M	III
11	Symptomatic	47	F	IV
12	Symptomatic	55	-	IV
13	Symptomatic	49	F	IV

CTRL = Healthy controls; I = early HD; II = light HD symptoms; III = moderate HD symptoms; IV = strong HD symptoms.

### TGF-β1 is mainly expressed by astrocytes in HD brains

In order to identify what cell population was primarily implicated in the synthesis of TGF-β1 in brain tissues along HD course, immunohistochemical studies were conducted by using marker of specific cell types. First, we investigated the involvement of microglia by using the microglia-specific Ionized calcium-binding adaptor molecule 1 (Iba1). Our data showed no co-localization between Iba1 and TGF-β1 immunopositive cells in none of the neuropathological grades of HD brains (Additional file 6), suggesting therefore, a poor implication of this cell type.

**Figure 6:**
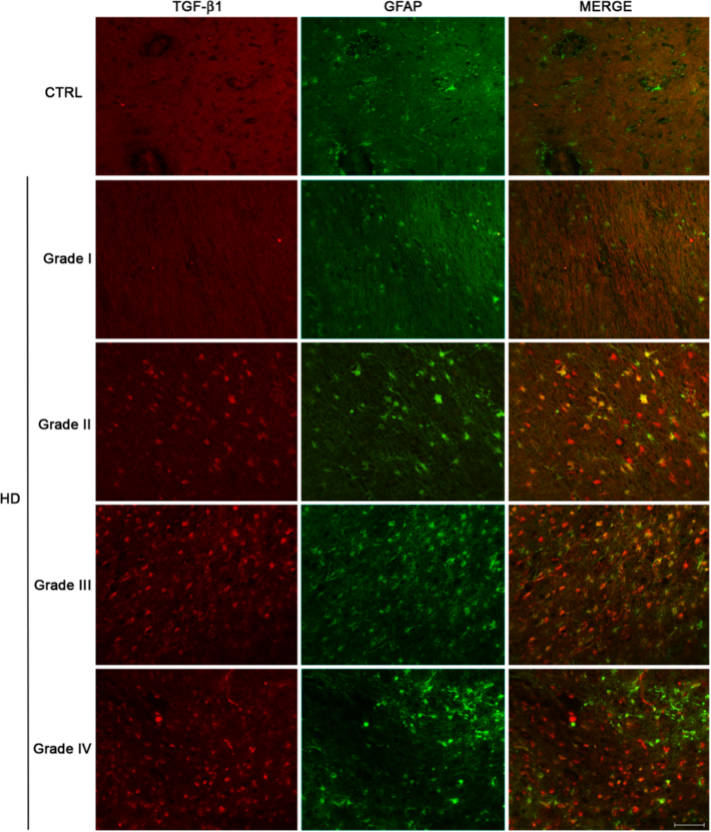
**Astrocytes are mainly involved in the synthesis of TGF-β1 in postmortem HD brains.** Representative microphotographs of double fluorescent staining for TGF-β1 and GFAP in post-mortem striatal tissues of control subjects and HD patients, showing co-localization between GFAP and TGF-β1 immunoreactive cells at different pathological grades (from II to IV).

Conversely, analysis of Glial Fibrillary Acid Protein (GFAP) immunoreactivity revealed a preferential implication of astrocytes in the synthesis of TGF-β1 in HD brains (Figure 6). GFAP immunoreactive cells showed co-localization with TGF-β1 positive cells starting from grade II HD patients up to later grades (Figure 6).

### Percentage of TGF-β1^+^ macrophages correlates with clinical and genetic parameters

**Figure 7:**
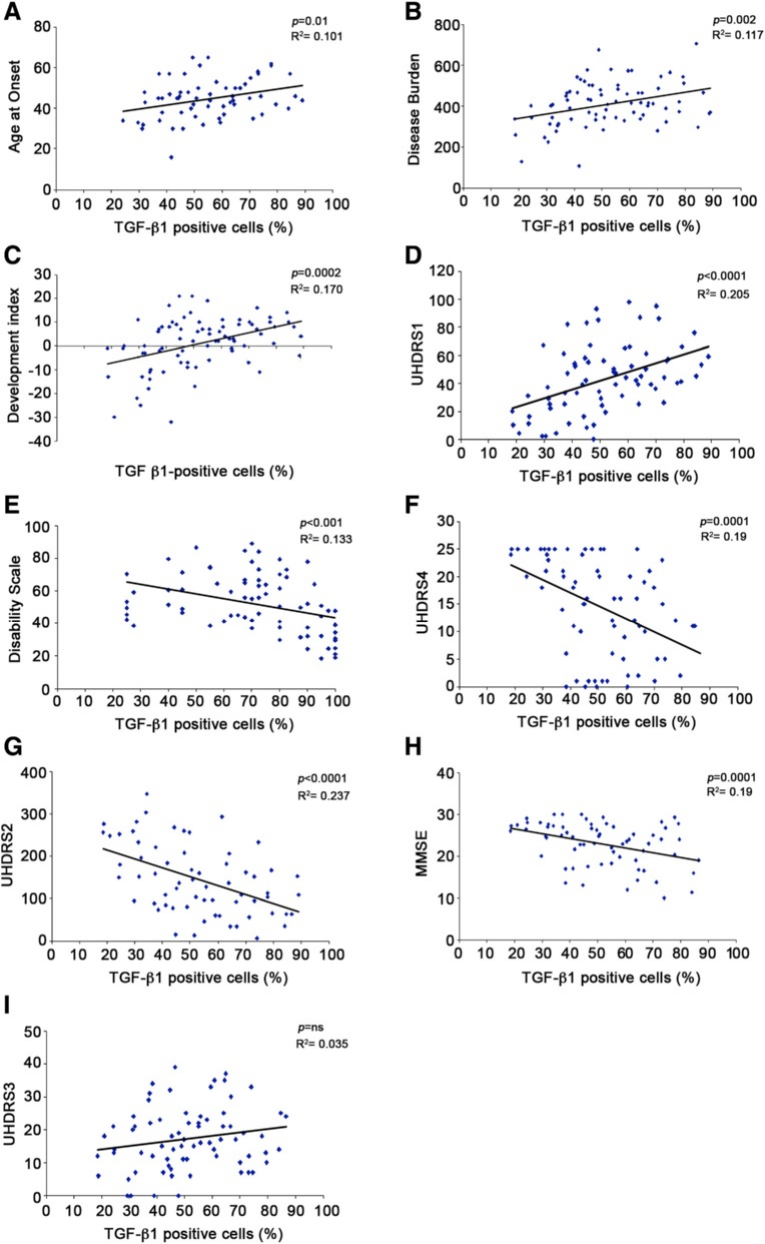
**Percentage of TGF-β1**^**+ **^**macrophages correlates with clinical and genetic parameters in HD individuals.** Linear regression analysis showing positive correlation between percentage of TGF-β1^+^ cells and age at onset (**A**, n = 69), disease burden (**B**, n = 76), HD development index (**C**, n = 79) and UHDRS1 (**D**, n = 72). Percentage of TGF- β1^+^ macrophages negatively correlates with disability scale (**E**, n = 74), UHDRS4 (**F**, n = 70), UHDRS2 (**G**, n = 64), MMSE (**H**, n = 67). No correlation was observed between percentage of TGF-β1^+^ macrophages and UHDRS3 (I, n = 72). Each dot represents a single subject.

When we explored possible relationships between TGF-β1 and clinical parameters, we observed a statistically significant positive correlation between the percentage of TGF-β^+^ cells and age at onset (Figure 7A), disease burden (Figure 7B), HD development index (Figure 7C), as well as motor performance score (UHDRS1) (Figure 7D). A significant negative correlation was also found with disability scale (Figure 7E), functional test scores (UHDRS4) (Figure 7F) and cognitive test scores (UHDRS2 and MMSE) (Figure 7G, H). Instead, we did not find any correlation between TGF-β1^+^ cells and the behavioural test score (UHDRS3) (Figure 7I).

## Discussion

Changes in the levels and the activities of endogenous neurotrophic factors are considered critical for the progression of degeneration in CNS diseases [28-30], including HD [31]. Defective bioavailability of such factors may have profound impact on the homeostasis of the brain, affecting neuroplasticity and leading to neuronal death [5]. Recently, we have reported dynamic variations of TGF- β1 levels throughout the natural course of the disease and argued that the defective bioavailability of the cytokine early in the disease might contribute to the development of HD [10].

In the present study, we show that changes of peripheral TGF-β1 levels may be attributable to an aberrant production by peripheral monocytes/macrophages. The number of TGF-β1-producing cells varied with disease progression and displayed a profile that was similar to the pattern of TGF-β1 levels in the serum of HD patients [10]. Therefore, lower number of TGF-β1^+^ monocytes/macrophages in pre-HD subjects, might potentially explain the reduced bioavailability of TGF-β1 in the serum of HD individuals at similar clinical stage. Both peripheral monocytic and macrophagic cell subsets from pre-HD subjects showed a significant reduction of TGF-β1^+^ cells when compared to controls and late stage HD patients. The lower percentage of TGF-β1^+^ monocytes-derived macrophages was associated with an increased intracellular content of the cytokine in pre-HD subjects and was accompanied by remarkable increase in TGF-β1 gene expression, suggesting an attempt of cells to counteract the early defective production of the trophic factor.

Macrophages represent a heterogeneous cell population that exhibits remarkably plasticity and can change functional state in response to micro-environmental cues [32]. Based on their activation state, macrophages can be divided into two polarized phenotypes known as M1, or “classical activated” and M2 or “alternative activated” macrophages [25,33]. Identification of distinct macrophages subsets with divergent effects is based on the expression of transcription factors, cell surface markers that provide a mechanism for their differential recruitment in response of different signal and production of specific cytokines [27,34].

M1 macrophages, also designed as CCR2^+^CX3CR1^-^ cells, exhibit a typical inflammatory phenotype and secrete high levels of pro-inflammatory cytokines including interleukin (IL)-6 and IL-12. Conversely, M2 macrophages or CCR2^-^CX3CR1^+^ cells exhibit anti-inflammatory and tissue-repair function and proficiently produce high amount of anti-inflammatory cytokines such as TGF-β1 and IL-10 [34-36].

Interestingly, a more detailed analysis of TGF-β1^+^ macrophages in HD revealed phenotypic heterogeneity of these cells at different stages of the disease. In particular, we found that a large number of inflammatory M1 macrophages dominated the early stage of the disease at the expenses of M2 macrophages; a phenomenon that was reversed later, in symptomatic HD patients. Moreover, analysis of the percentage of either IL-12 or IL-10-producing cells further support macrophages heterogeneity in HD. IL-12^+^ macrophages were significantly more numerous in pre-HD subjects than they were in symptomatic HD patients, who instead showed a remarkable increase of percentage of IL-10^+^ macrophages. Changes in the percentage of both cytokines-producing cells would explain the disease stage-dependent variation of cytokines levels in HD individuals [2]. While increased percentage of IL-12^+^ macrophages (M1) may explain the inflammation state in the early stage of the disease, elevated IL-10 producing macrophages (M2) in advanced disease stage may enhance an adaptive immune response, convey neuroprotective signals and, possibly, outline a potential repairing attempt of tissues in HD. Yet, the mechanisms that govern macrophage polarization to different phenotypes remain to be defined, however, our results suggest that it could be mediated by a variable activity of NF-κB.

NF-κB is widely known for its role in the regulation of inflammation and immune response [37]. It is an inducible heteromeric transcription factor classically composed of p50 and p65 subunits. While p65 possesses a transactivation domain and modulates most of the NF-κB’s transcriptional activity, p50 does not [38,39]. Activation of NF-kB promotes M1 macrophage polarization and turns inflammation processes on [40], while its inhibition results in switching the inflammation processes off [41-43]. Dysregulation of NF-κB activity has been implicated in the pathogenesis of multiple diseases such as inflammatory diseases and neurodegenerative disorders [44] including HD [45,46].

Here, we found that NF-κB pathway changed along HD course in both central and peripheral district with similar pattern. Unlike a relatively high expression of NF-κB -p65 early in the disease, late HD patients showed levels of NF-κB -p65 reduced compared to early HD subjects and similar to healthy controls. Whether the reduction of NF-κB -p65 in HD patients is due to its selective degradation [47] or depends on anti-inflammatory strategy that macrophages can adopt to counteract the overproduction of inflammatory cytokines needs to be further investigated. Predominance of classical NF-κB heterodimer p50/p65 promotes M1 polarization, whereas M2 polarization is selectively mediated by p50/p50 homodimers [38]. NF-κB-p50 plays a crucial role in the control of M1-vs. M2-driven inflammation by selectively promoting the production of the anti-inflammatory cytokine IL-10 [48] that, in turns promotes the formation of p50/p50 homodimer [49] and inhibits NF-κB activity [38].

Based on this evidence, we hypothesized that the lower availability of NF-κB -p65 late in the disease could correlate with a preferential formation of p50/p50 homodimers thereby driving anti-inflammatory IL-10 gene transcription and subsequently favoring M1-M2 switch. Further studies, however, are needed to address this specific issue. Although little is known about the acquisition and maintenance of M2 phenotype, we believe that M1-M2 switch in HD, in all probability, points out differential roles of peripheral immune cells in the disease induction or progression and may provide protection against overwhelming uncontrolled inflammation. However the underlying molecular mechanism remains to be elucidated. Ongoing studies in our laboratory are examining whether and how mHtt can influence monocytes-derived macrophages polarization along disease course.

Furthermore, similarly to the periphery, the ability of cells to produce TGF-β1 in the brain varied during disease course, indicative of a possible parallelism between peripheral dysfunction and central defects. TGF-β1 immunoreactive cells were merely detectable in the pathological grade I HD brains and increased dramatically with the severity of pathological grades. TGF-β1 immunoreactivity was paralleled by a gradual increase in GFAP immunopositive reactive astrocytes, indicating a predominant role of these cells to synthesize the neurotrophin in HD brains and highlighting a spread reactive gliosis, a coordinated cellular response usually aimed at mitigating damage to nearby neurons [50]. A phenomenon this, which could be compared to what occurs in periphery, where anti-inflammatory pattern dominates the late clinical stage of the disease. Since the biological effects of TGF-β1 are diverse, the pathological significance of both clinical stage-dependent changes of TGF-β1 content in periphery and pathological grade-dependent changes in post-mortem brain tissues of HD patients is thought to be complex and, further studies are needed to specifically address this issue.

Interestingly, changes in the TGF-β1^+^ macrophages number correlated with HD clinical features, raising the hypothesis that peripheral TGF-β1 may represent a potentially valuable parameter for monitoring disease development.

## Conclusions

In summary our study suggests that alteration in monocytes/macrophages homeostasis plays a critical role in establishing the defective production of TGF-β1 in HD and highlights an interesting parallelism between peripheral dysfunction and central defect. We believe that the discovery of macrophages plasticity and an unbalanced M1/M2 phenotype in HD point out a novel biological process that could explain the variable inflammatory profile in HD and eventually define the possible molecular mechanism underlying immune response in the disease. Macrophages heterogeneity in HD likely reflects dynamic variation in the micro-environmental changes during the transition from early to advanced HD stages, which would result in progressive modulation of NF-κB activity in macrophages and their subsequent conversion from M1 to M2 phenotype.

To our knowledge, this is the first evidence of a biological phenomenon never described before in HD. Understanding the biological mechanisms whereby each of the macrophages subset is induced to assume these different roles may provide new opportunities to therapeutically manipulate immune response in HD.

In conclusion, we believe that our study may be of clinical relevance as it has the potential of leading to the identification of possible indicator for predicting HD onset that could advance the design of clinical trials to delay onset or slow progression in HD.

## Methods

### Subjects

A total of 112 HD subjects (14 pre-HD, 15 stage I, 36 II, 47 III-V), and 46 gender- and age-matched healthy controls divided into 3 groups: 25–40, 41–55, and 55–80 year-old were recruited. Subjects’ demographic, clinical and genetic characteristics are reported in Table 1. Pre-HD subjects had previously requested a pre-symptomatic genetic test by entering a specific program whose protocol was ethically approved [51]. All HD subjects revealed a CAG repeat expansion mutation and all of them as well as controls were required to sign an informed consent before recruitment in the study. All human experiments were performed in accordance with the Declaration of Helsinki [52].

Control subjects with a suspect of cardiovascular, psychiatric or neurodegenerative disorders other than HD, were excluded from this study. Clinical examinations were conducted using the Unified Huntington’s Disease Rating Scale (UHDRS) to measure motor, cognitive, behavioural and general function [53] and the Mini-Mental State Examination (MMSE) was used to screen general cognitive function [53,54]. Pre-HD subjects included either asymptomatic individuals (total motor score of < 5 in the UHDRS and cognitive and behavioural assessment within the normality) or individuals with soft signs (suspicious clinical features that were insufficient to warrant a diagnosis of HD) [55]. The patients’ age at onset (AO) was retrospectively established by interviews to family members regarding the first neurological manifestations [56], thus excluding, in this first study stage, subjects showing severe cognitive and psychiatric abnormalities that represented a permanent change from the normal state [57]. The predicted years to manifest the disease were calculated on the basis of the survival analysis formula described by Langbehn et al. (2004) [58]. To estimate the progression of the pathological process from pre-HD stage, we calculated the ‘HD development’ index by combining the predicted years to onset for pre-HD subjects and disease duration (years-from-onset) for patients [59,60]. The disease burden (DB) index was measured according to the previously described formula: age x (CAG–35.5) [61]. The Disability Scale (DS) combines patients’ independence and motor performance, thus taking into account the subjects’ independence on neurological motor impairment [62]. The disease stage was calculated according to the Total Functional Capacity (TFC) score [63].

### Human macrophages

#### Isolation and differentiation

Peripheral blood mononuclear cells (PBMCs) were obtained by density gradient centrifugation (Ficoll–Hypaque) from freshly drawn venous blood (platelet-free). The cell pellet was layered on a Percoll (GE Healthcare) gradient and the interphase containing monocytic cells was obtained following a 800 *g* centrifugation [64]. After washing monocytes were seeded into 24-well plates culture containing RPMI (Sigma, San Louis, MO, USA) supplemented with 10% heat inactivated foetal bovine serum (FBS), 2 mM L-glutamine and 100 μg/ml streptomycin and 100 units/ml penicillin. Cells were maintained for 7 days at 37°C in a humidified atmosphere containing 5% CO_2_ and cultured in presence of 100 ng/ml of macrophages colony stimulating factor (M-CSF, GE-Healthcare) to obtain fully differentiated macrophages [65].

#### Flow cytometry analysis

Adherent macrophagic cells were detached by vigorous pipetting ice-cold phosphate buffered saline (PBS), centrifuged at 200 *× g* for 5 minutes and washed with PBS. Cell suspension was then fixed with 4% PFA and incubated with FITC-conjugated anti-human CD80 (EuroBioSciences GmbH, Germany) or an equivalent amount of isotypic control IgG1 (EuroBioSciences GmbH, Germany) for 1 h. CD80-positive macrophages were >90% gated cells (data not shown). For the detection of intracellular cytokines, cells were permeabilized with a buffer containing 0.1% (w/v) tritonX-100, 0.05% (w/v) NaN_3_ in PBS, and incubated for 1 h at room temperature with PE-conjugated anti-human TGF-β1 or PE-conjugated anti-human IL-10 or anti-human IL-12 (BD Pharmingen, San Jose, CA USA) or an equivalent amount of IgG1 and IgG2a (BD Pharmingen, San Jose, CA USA) isotypic controls, respectively. Cells were washed and re-suspended with PBS before acquisition. The cytokine-positive cells and the relative mean fluorescence unit (MFU) were scored on the basis of isotype controls.

### Real-time RT-PCR analysis

TGF-β1 gene expression in macrophages from pre-HD subjects, HD patients and healthy controls, was measured by quantitative PCR analysis performed on a StepOnePlus instrument (Applied Biosystems) by using the following primer sequences: Fwd: 5′-CGAGCCTGAGGCCGACTACTA-3′; Rev: 5′-CTCGGAGCTCTGATGTGTTGAA-3′. Briefly, total RNA was extracted using RNeasy kit (Qiagen) according to the manufacturer’s instructions and reverse transcribed using Superscript II reverse transcriptase (Invitrogen) and oligo-d(T) primer. Resulting cDNAs were amplified using Power SYBR Green PCR Master Mix (Applied Biosystems) following the manufacturers’ instructions. The level of each mRNA was normalized to that of cyclophilin A (CypA). PCR cycling parameters were as follows: 50°C for 2 min, 95°C for 5 min, followed by 40 cycles of 95°C for 20 s, 60°C for 1 min, and 72°C for 40 s.

### Cell subsets from whole blood

Whole blood analyses were carried out in 81 HD individuals (5 pre-HD subjects, 11 I HD, 36 II HD and 29 III-V HD stage patients) and 26 gender- and age-matched healthy controls. Venous blood drawings were collected applying minimal venous stasis in order to minimize cell activation. Whole blood (50 ml) for each sample was fixed with PFA 2% over night at 4°C and circulating cell subsets were distinguished from each other on the basis of physical parameters by using forward (FSC-H, cell volume index) and side light-scatter patterns (SSC-H, cell density index). Cells were then labelled with FITC-conjugated anti-human CD14 (BD Pharmingen, San Josè, CA USA) or equivalent amount of isotypic control IgG2a, k (BD Pharmingen, San Josè, CA USA) for the detection of monocytes. CD14-positive monocytes were >90% gated cells (data not shown). After washing with PBS, cells were permeabilized with 0.3% tritonX-100 and incubated with PE-conjugated anti-human TGF-β1 (R&D Systems, Minneapolis, MN USA) or an equivalent amount of isotypic control IgG1 (R&D Systems, Minneapolis, MN USA). The percentage of TGF-β1^+^ cells and the intracellular content of the cytokine (MFU), were scored on the basis of isotype control. All the experiments were performed by using a Becton-Dickinson FACS Calibur flow cytometer (BD, San Josè, CA, USA).

### Flow cytometry analysis of macrophage M1 and M2 subsets

Analyses of macrophage subsets were carried out in 12 HD individuals (5 early HD subjects and 7 II-III HD stage patients) and 5 gender and age-matched healthy controls. After isolation and differentiation, detached monocytes-derived macrophages were incubated with FITC-conjugated anti-human CCR2 and with PE-conjugated anti-human CX3CR1 or equivalent amount of isotypic control IgG2b and IgG1, respectively. Cells were incubated for 45 minutes at 4°C in the dark, washed with PBS and acquired to the cytometer. Macrophages were gated on the basis of cells size (FSC-H) and cells density (SSC-H). The percentage of M1 macrophages was identified as CCR2 positive-CX3CR1 negative cells (CCR2^+^CX3CR1^-^) while the percentage of M2 macrophages was identified as CCR2 negative -CX3CR1 positive cells (CCR2^-^ CX3CR1^+^) on the basis of isotype controls. All antibodies were purchased from R&D Systems (Minneapolis, MN USA). All the experiments were performed by using a Becton-Dickinson FACSCalibur flow cytometer (BD, San Josè, CA, USA).

### Immunoblotting

Monocytes-derived macrophages from pre-HD subjects, symptomatic HD patients and controls were lysed in lysis buffer containing 20 mM Tris, pH 7.4, 1% Nonidet P-40, 1 mM EDTA, 20 mM NaF, 2 mM Na3V04, and 1:1000 protease inhibitor mixture (Sigma-Aldrich), sonicated with 2 × 10 s pulses and then centrifuged for 10 min at 10,000 × g. For analysis of NF-κB-p65 protein levels, total protein lysate was immunoblotted with anti-NF-κB-p65 (C-20) (1:500; Santa Cruz, sc-372). Ponceau Red staining served as a loading control [66]. TGF-β1 expression was determined by using monoclonal anti- TGF-β1 (T0438) (1:1000; Sigma-Aldrich). A goat polyclonal anti-Talin (C-20; Santa Cruz, sc-7534) was used as loading control for normalization. HRP-conjugated polyclonal secondary antibody (GE-Healthcare) was used at 1:5000 dilution. Protein bands were detected by ECL Prime (GE Healthcare) and quantitated with Quantity One (Bio-Rad Laboratories) and/or ImageJ software.

### TGF-β1 in human post-mortem brain samples

Post-mortem brain tissues from ten patients at different pathological grades of HD [67] and three healthy controls were examined in this study. Samples were obtained by the New York Brain Bank at Columbia University, New York, USA. Clinical and neuropathological data were summarized in Table 2. Formalin fixed, paraffin-embedded striatal tissues were sectioned at 10 mm. Deparaffinized sections were soaked in 3% hydrogen peroxide to block endogenous peroxidase activity. Sections were treated with Pronase at 37°C for 10 min for antigen retrieval and incubated overnight with monoclonal mouse anti-TGF-β1 antibody (1:1000; Chemicon, CA). TGF-β1 expression was detected by incubating the sample for 1 hour with secondary biotinylated anti-mouse antibody (1:200; Vector Laboratories, Burlingame, CA). Visualization of the immunoreaction was performed with 0.05% 3,3′-diaminobenzidine tetrachloride (DAB) (ABC Elite kit; Vector Laboratories). Control staining was performed without the specific primary antibody. Double fluorescence immunohistochemistry was performed by incubating brain sections over-night with polyclonal rabbit anti-TGF-β1 antibody (1:100; Sigma-Aldrich) and monoclonal mouse anti-GFAP (1:300; Sigma-Aldrich) or polyclonal goat anti-Iba1 (1:2000; Abicam). Proteins were then visualized after 1 hour of incubation with secondary Cy3 anti-rabbit (1:200; Chemicon), and fluorescein anti-mouse (1:100; Vector Laboratories) or biotin anti-goat (1:200; Vector Laboratories) and fluorescein anti-biotin (1:100; Vector Laboratories) antibodies.

### Statistical analysis

ANOVA followed by the Tukey’s multiple comparisons test was used for the analysis of data with more than two groups. Linear dependence of TGF-β1^+^ macrophages on Age at Onset (AO), Disease Burden (DB), Disability Scale (DS), Time from/to Onset (TO), UHDRS1, 2, 3, 4 scores and MMSE was determined by a simple regression model. Data were considered statistically significant at *p* < 0.05. Statistical analysis was performed with Biostat2009 software.

## Abbreviations

AO: Age at onset; BDNF: Brain derived neurotrophic factor; CAG: Cytosine adenine guanine; CCR2: C-C chemokine receptor type 2; CD14: Cluster differentiation 14; CD14: Cluster differentiation 80; CNS: Central nervous system; CTRL: Healthy control; CypA: Cyclophilin A; CX3CR1: CX3C chemokine receptor 1; DAB: 3*,*3*′-*diaminobenzidine tetrachloride; DB: Disease burden; DS: Disability scale; FACS: Fluorescence activated cell sorting; FBS: Fetal bovine serum; FITC: Fluorescein isothiocyanate; GDNF: Glia derived neurotrophic factor; GFAP: Glial fibrillary acid protein; HD: Huntington disease; Htt: Huntingtin; Iba1: Ionized calcium-binding adaptor molecule 1; IL-10: Interleukine-10; IL-12: Interleukine-12; IL-6: Interleukine-6; M-CSF: Monoclonal colony stimulating factor; mHtt: mutant Huntingtin; MFU: Mean fluorescence unit; MMSE: Mini mental state examination; NF-κB: Nuclear factor kappa-light-chain-enhancer of activated B cells; PBMC: Peripheral blood mononuclear cells; PBS: Phosphate-buffered saline; PFA: Paraformaldehyde; preHD: pre-manifested subjects; polyQ: polyglutamine; RPMI: Roswell park memorial institute medium; s.d.: standard deviation; TFC: Total functional score; TGF-β1: Trasforming growth factor beta-1; UHDRS: Unified Huntington disease rating scale; yrs: years.

## Competing interests

The authors declare no competing financial interests.

## Authors’ contributions

ADP, VM, GB, FN and FS contributed to project design, data analyses and paper writing. SA performed the majority of the experiments. EA, FE and CLB provided technical assistance. EC and JPV provided post-mortem brain samples and assistance with classification of neuropathological grade. All authors have read and approved the final manuscript.

## Supplementary Material

Additional file 1 **Total number of whole blood cells did not vary between HD individuals and healthy controls.** A-C, Bar histograms showing total number of lymphocytic, granulocytic and monocytic cells in both HD individuals (n = 81, gray bars) and healthy control subjects (n = 26, white bar). Whole blood cell populations were distinguished from each other on the basis of physical parameters by using forward (FSC-H, cell volume index) and side light-scatter patterns (SSC-H, cell density index). Cells number is expressed as n° cells × 10^3^/ml. Data are shown as mean ± s.d. Click here for file

Additional file 2 **Representative flow cytometric histograms showing TGF-β1**^**+**^**monocytes in control subject (CTRL), pre-manifested subject (preHD) and stage HD patients (HD).** TGF-β1^+^ cells were identified in FL-2^+^ fluorescence scatter (black histogram). Isotype control (gray histogram) was used to determine the 95% confidence interval of nonspecific fluorescence. Click here for file

Additional file 3 **Immunoblotting analysis showing changes of TGF-β1 expression at different disease stages.** Representative immunoblot (top) and densitometric analysis (bottom) of TGF-β1expression in healthy controls (CTRL, n = 4), pre-manifested subjects (pre-HD, n = 4) and severe HD patients (HD, n = 4). Bar graph represents the mean values ± s.d. * *p* < 0.05 (ANOVA followed by Tukey’s multiple comparisons test). Click here for file

Additional file 4 **Representative flow cytometric histograms showing TGF-β1**^**+**^**macrophages in control subjects (CTRL), pre-manifested subjects (pre-HD) and stage HD patients (HD).** TGF-β1^+^ cells were identified in FL-2^+^ fluorescence scatter (black histogram). Isotype control (gray histogram) was used to determine the 95% confidence interval of nonspecific fluorescence. Click here for file

Additional file 5 **Bar histograms showing no-age related changes in the percentage of TGF-β1**^**+**^**macrophages in control subjects.** A, Bar histograms showing percentage of TGF-β1^+^ macrophages in healthy controls divided into age groups (25–40, 41–55, 56–80). B, Bar histograms showing no changes of TGF-β1 content (MFU) in macrophages, from the same control individuals. Data are shown as mean ± s.d. Click here for file

Additional file 6 **Microglia appear not to be implicated in the synthesis of TGF-β1 in post-mortem brain tissues along HD course.** Representative microphotographs of double fluorescent staining for TGF-β1 and Iba1 in post-mortem striatal tissues of control subjects (CTRL), and HD patients at different pathological grades (from I to IV). No colocalization between Iba1 immunoreactive cells and TGF-β1 immunopositive cells in none of the pathologically graded brains was observed. Click here for file
