# Supplementary figures and images for: Changes of peripheral TGF-β1 depend on monocytes-derived macrophages in Huntington disease
Source: Mol Brain. 2013 Dec 13;6:55. doi: 10.1186/1756-6606-6-55 (PMC4029620; doi:10.1186/1756-6606-6-55)

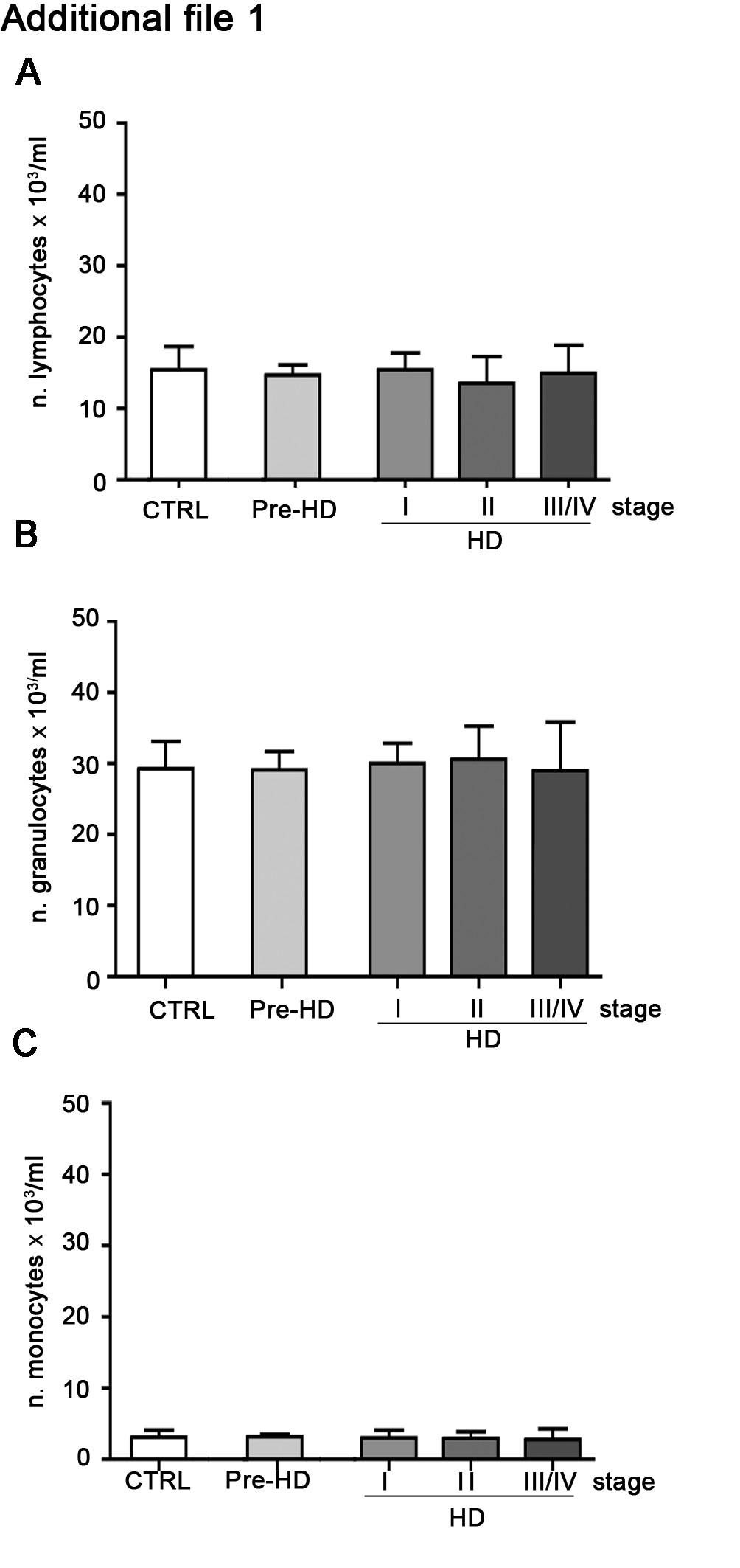

Supplement: Additional file 1 — Total number of whole blood cells did not vary between HD individuals and healthy controls. A-C, Bar histograms showing total number of lymphocytic, granulocytic and monocytic cells in both HD individuals (n = 81, gray bars) and healthy control subjects (n = 26, white bar). Whole blood cell populations were distinguished from each other on the basis of physical parameters by using forward (FSC-H, cell volume index) and side light-scatter patterns (SSC-H, cell density index). Cells number is expressed as n° cells × 103/ml. Data are shown as mean ± s.d. [file 1756-6606-6-55-S1.tiff]

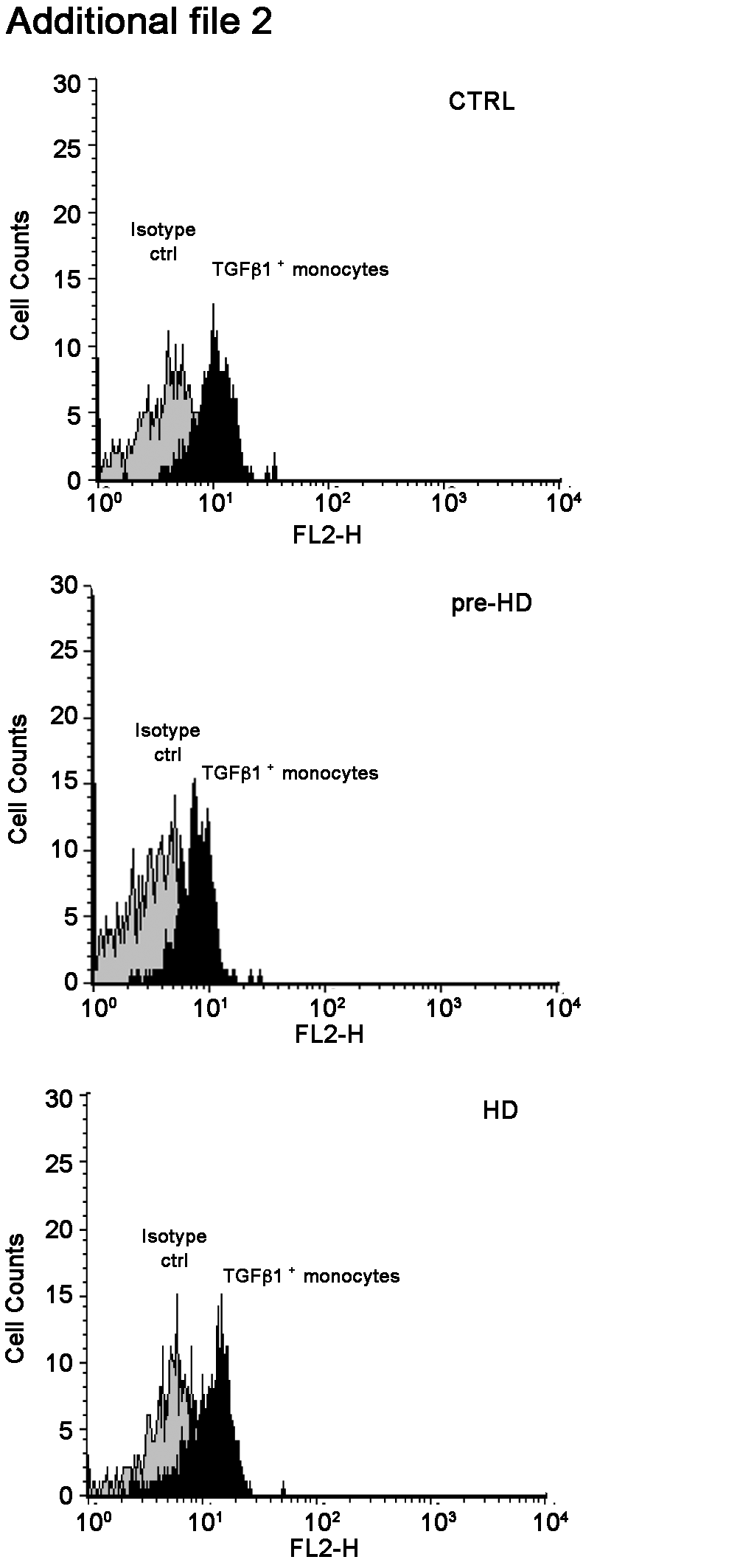

Supplement: Additional file 2 — Representative flow cytometric histograms showing TGF-β1+monocytes in control subject (CTRL), pre-manifested subject (preHD) and stage HD patients (HD). TGF-β1+ cells were identified in FL-2+ fluorescence scatter (black histogram). Isotype control (gray histogram) was used to determine the 95% confidence interval of nonspecific fluorescence. [file 1756-6606-6-55-S2.tiff]

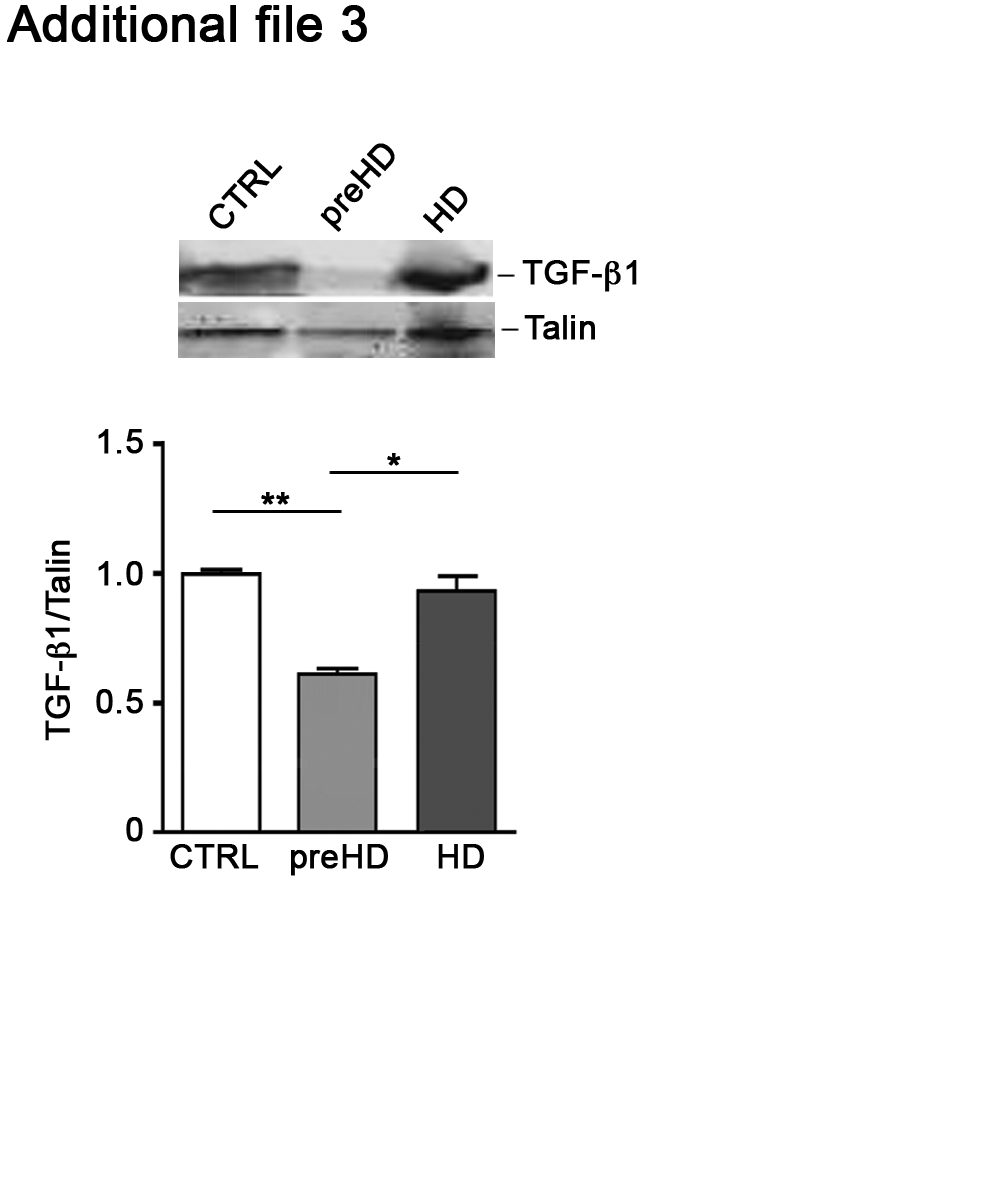

Supplement: Additional file 3 — Immunoblotting analysis showing changes of TGF-β1 expression at different disease stages. Representative immunoblot (top) and densitometric analysis (bottom) of TGF-β1expression in healthy controls (CTRL, n = 4), pre-manifested subjects (pre-HD, n = 4) and severe HD patients (HD, n = 4). Bar graph represents the mean values ± s.d. * p < 0.05 (ANOVA followed by Tukey’s multiple comparisons test). [file 1756-6606-6-55-S3.tiff]

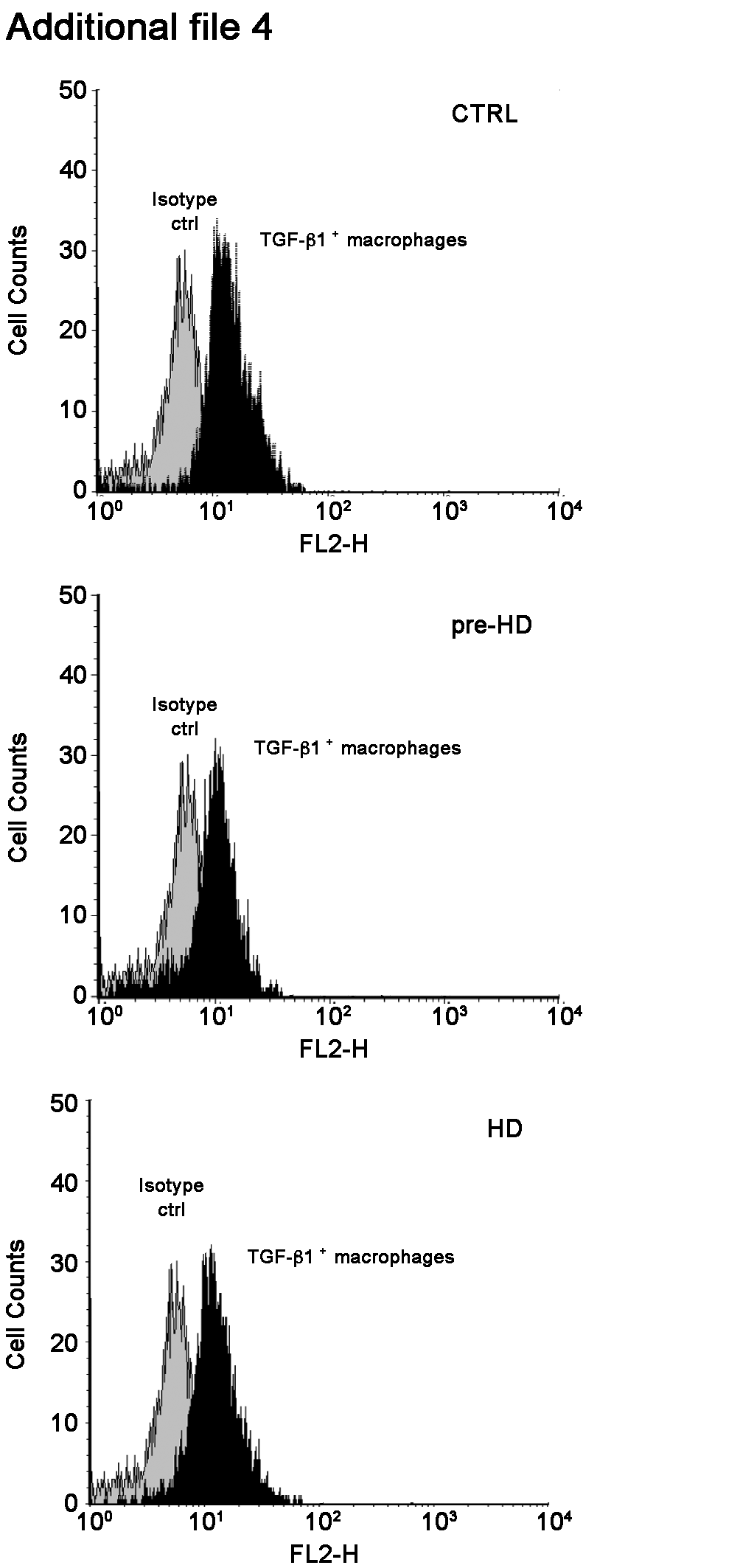

Supplement: Additional file 4 — Representative flow cytometric histograms showing TGF-β1+macrophages in control subjects (CTRL), pre-manifested subjects (pre-HD) and stage HD patients (HD). TGF-β1+ cells were identified in FL-2+ fluorescence scatter (black histogram). Isotype control (gray histogram) was used to determine the 95% confidence interval of nonspecific fluorescence. [file 1756-6606-6-55-S4.tiff]

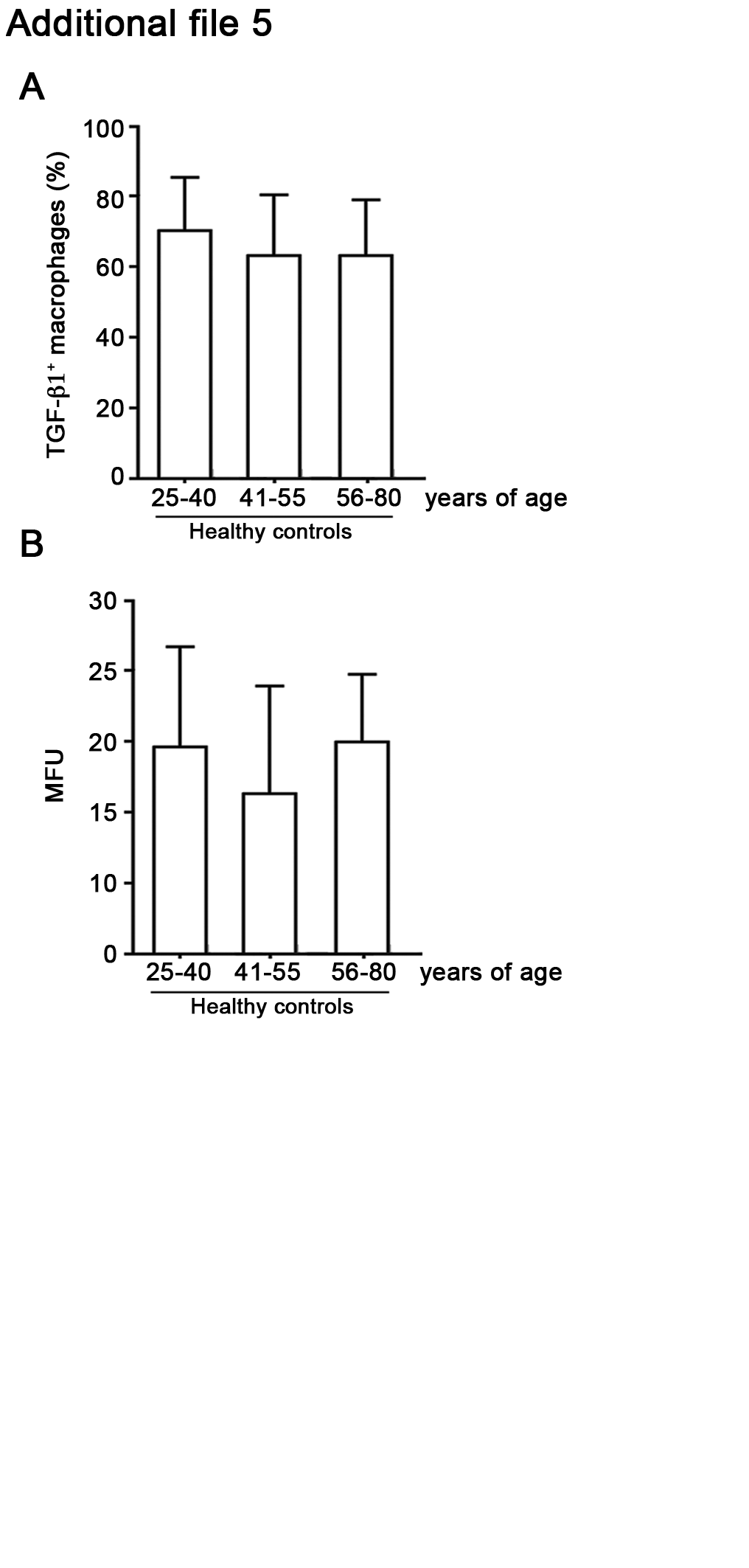

Supplement: Additional file 5 — Bar histograms showing no-age related changes in the percentage of TGF-β1+macrophages in control subjects. A, Bar histograms showing percentage of TGF-β1+ macrophages in healthy controls divided into age groups (25–40, 41–55, 56–80). B, Bar histograms showing no changes of TGF-β1 content (MFU) in macrophages, from the same control individuals. Data are shown as mean ± s.d. [file 1756-6606-6-55-S5.tiff]

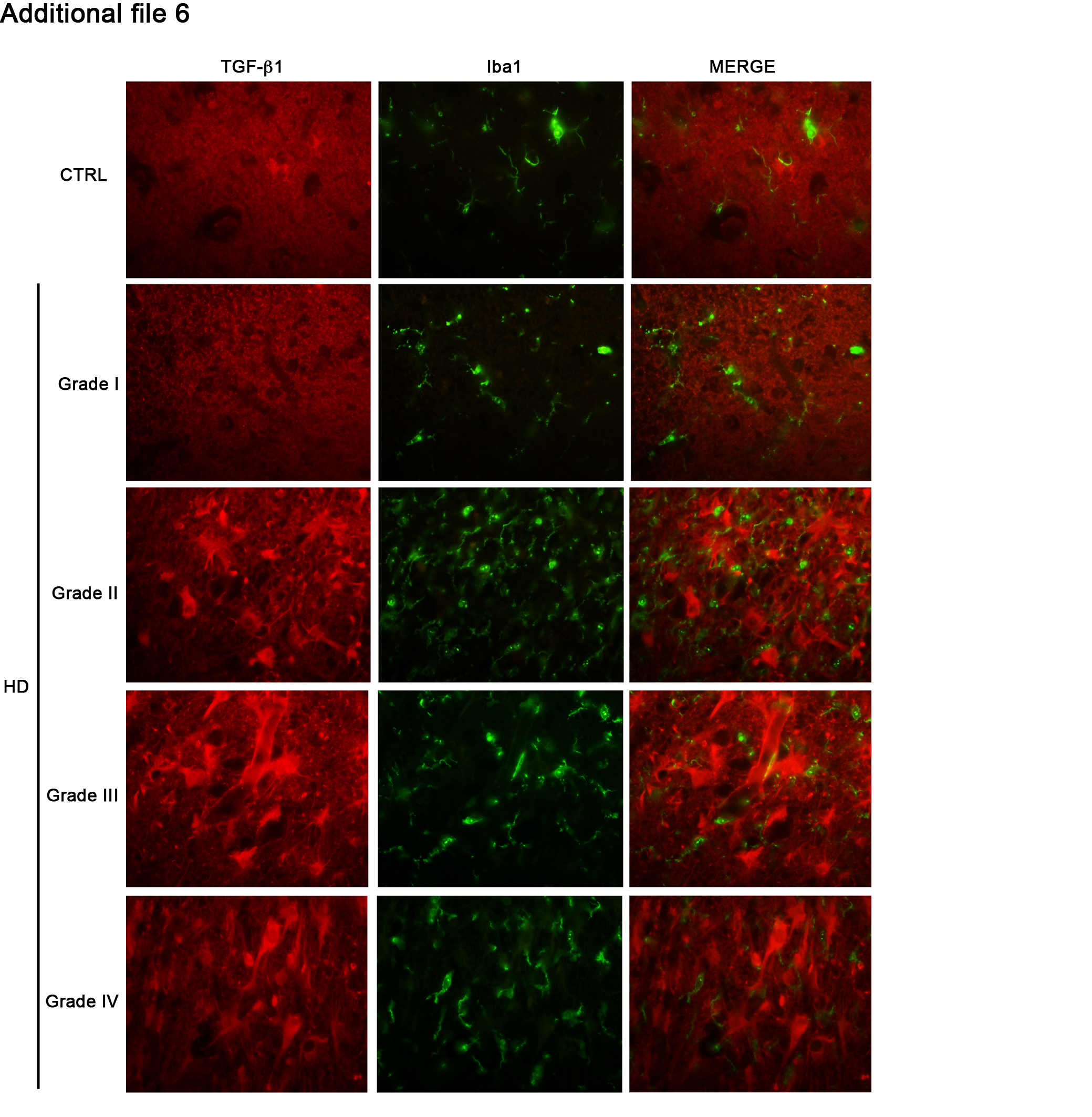

Supplement: Additional file 6 — Microglia appear not to be implicated in the synthesis of TGF-β1 in post-mortem brain tissues along HD course. Representative microphotographs of double fluorescent staining for TGF-β1 and Iba1 in post-mortem striatal tissues of control subjects (CTRL), and HD patients at different pathological grades (from I to IV). No colocalization between Iba1 immunoreactive cells and TGF-β1 immunopositive cells in none of the pathologically graded brains was observed. [file 1756-6606-6-55-S6.tiff]
